# Determining behavioral proxies of preference: mate choice and the New England Cottontail (*Sylvilagus transitionalis*)

**DOI:** 10.1093/jmammal/gyag023

**Published:** 2026-07-09

**Authors:** Hannah Petit, Vivian Stansell, Louis Perrotti, Justin T Richard

**Affiliations:** Department of Fisheries, Animal and Veterinary Sciences, University of Rhode Island, Kingston, 9 East Alumni Ave, RI 02881, United States; Department of Fisheries, Animal and Veterinary Sciences, University of Rhode Island, Kingston, 9 East Alumni Ave, RI 02881, United States; Conservation Programs, Roger Williams Park Zoo, Providence, RI 029017, United States; Department of Fisheries, Animal and Veterinary Sciences, University of Rhode Island, Kingston, 9 East Alumni Ave, RI 02881, United States

**Keywords:** female mate choice, lagomorph, reintroduction program, reproductive ecology

## Abstract

Reintroduction breeding programs are a key conservation strategy that can be more successful when breeding pairs are determined through mate choice. The vulnerable New England Cottontail (NEC) (*Sylvilagus transitionalis*) has lost much of its historic range, prompting the establishment of a program to release zoo-bred rabbits to bolster declining populations. To improve offspring production, a mate choice experiment tested the preference of 8 females with 3 males in a pen that allowed visual and olfactory contact with all males simultaneously. The length of time, frequency of visits, female urine markings, number of barrier investigations, and female proximity to each of the males were recorded as potential mate preference proxies. Females were then paired with each of the 3 males during the breeding season (*n *= 24 pairings). The rate at which females visited males during the preference test trials was positively correlated with the occurrence of mounting behavior. Compared with males least visited during the preference trial, females were 2.5 times more likely to produce offspring with males that they visited the most. Visit frequency during a preference test trial can therefore be used as a proxy for female preference in future breeding seasons. This study highlights the significance of incorporating mate selection into breeding programs and contributes to our understanding of NEC ecology.

## Teaser

Conservation breeding programs are most effective when animals can express natural behaviors. The New England Cottontail (NEC) (*Sylvilagus transitionalis*), a vulnerable rabbit, relies on zoo-based breeding programs to support wild populations. To test whether mate choice could improve reproductive success, researchers observed how females interacted with 3 males simultaneously, recording time spent nearby, repeated visits, and scent marking. Females were more than twice as likely to produce offspring with the males they visited most often, and visit frequency strongly predicted the occurrence of mounting. These results show that simple preference tests provide a practical way to boost breeding outcomes. Incorporating mate choice into conservation programs may strengthen efforts to restore the NEC and other threatened species.

Reintroduction breeding programs are an important conservation strategy that aims to bolster wild populations by releasing captive-bred individuals. These breeding programs have been instrumental in the recovery of various species, such as black-footed ferrets (*Mustela nigripes*) and Columbia Basin pygmy rabbits (*Brachylagus idahoensis*; Jachowski and Lockhart 2009; DeMay et al. 2017). Offspring production in these programs can be improved by including opportunities for mate choice, rather than providing only 1 potential mate. Mate choice is a reproductive strategy that leads to non-random mating when access to mating is determined by 1 sex, often the female, assessing the characteristics possessed by the opposite sex (Edward 2015). This strategy offers numerous fitness advantages, such as improved fecundity and higher-quality offspring (Andersson 1994), which have also been recorded in managed breeding programs (Martin-Wintle et al. 2019). For example, experiments incorporating female mate choice in conservation breeding programs for eastern barred bandicoots (*Perameles gunnii*) and stripe-faced dunnarts (*Sminthopsis macroura*) have resulted in better reproductive performances amongst preferred pairings (Harnett et al. 2018; Parrot et al. 2019). The increase in breeding success can be quite substantial. For instance, 75% of giant pandas (*Ailuropoda melanoleuca*) paired based on preference produced cubs compared with 0% of non-preferred pairings (Martin-Wintle et al. 2015). Mate choice has also been associated with reduced aggression between mates, an important concern for the long-term viability of any given individual in a breeding program (Roberts and Gosling 2004). These studies highlight the importance of determining preference before pairing animals in breeding programs to maximize reproductive success.

However, determining preferred traits that are actively selected in this context is not always straightforward. Selected characteristics vary between species and even among individuals from the same species, including morphological, behavioral, or biochemical phenotypes (Dougherty 2020). This variability is influenced by the environment, experiences, and current physiological state of an animal (Dougherty 2020). To reduce aggression and ensure that the identity of the sire is known, many breeding programs cannot logistically create free-choice mating opportunities as might occur in the wild. Instead, many mate choice studies use a proxy measure of preference observed during trials in which the chooser is presented with multiple potential mates through physical barriers that prevent mating. Proxy measures include the length of time spent near a potential mate (Fisher et al. 2003), relative proximity to potential mates (Martin and Shepherdson 2012), or specific behaviors performed near the males, such as scent marking and urination (Witte 2006; Martin and Shepherdson 2012). Using proxy measures of preference allows for a variety of male stimuli to be used in preference assessments, from scent exposure to limited physical contact (Dougherty 2020). Proxy measures also make it possible to investigate preference with smaller sample sizes: instead of measuring whether females choose to mate or not mate with a single male, they can be exposed to a variety of males to determine desirable traits in multiple potential mates. These measures may not only signify individual preference but may also indicate preference at the population level if some males are consistently preferred by multiple females.

To ensure that proxy measures accurately reflect choice, a preference test is performed, followed by pairing individuals for breeding. Mating and other measures of reproductive success can then be compared with the proxy measures observed during the preference test. Observations of mating are required to validate proxy measures, as only then does mate choice impact reproductive outcomes (Dougherty 2020). A correlation between the proxy measure and reproductive outcomes indicates that the proxy measure accurately reflects preference. These predictive proxies could then be used to assess preference in future trials, simplifying the incorporation of mate choice into a breeding program. Given the impact that mate choice has had on managed breeding programs across a variety of taxa (Martin-Wintle et al. 2019), this approach has the potential to increase the efficiency, productivity, and conservation impact of the breeding program.

One species that could benefit from integrating mate choice into their breeding program is the New England Cottontail (NEC, *Sylvilagus transitionalis*). The NEC is listed as a vulnerable species by the International Union for Conservation of Nature (IUCN), with populations still declining (Litvaitis and Lanier 2019). Habitat destruction and fragmentation have left the NEC occupying less than 14% of its historic range (Litvaitis et al. 2006). Due to the concern for NECs, Roger Williams Park Zoo (RWPZ) created a reintroduction breeding program, which has reintroduced >250 kits to the states of Rhode Island, New Hampshire, Massachusetts, and Maine, United States (Brown and Puccia 2022). Pairings for breeding are determined through genetic considerations, but a majority of pairings do not result in kits. Continuous observations of 12 pairings in this program revealed behavioral differences between pairs that reproduce and those that do not, as well as variation within individual males and females when paired with different potential mates (Petit et al. 2024). In pairs that reproduce, males are more likely to follow behind females as they locomote, while in non-productive pairings, females are more likely to dash quickly past the male, precluding this following behavior. These observations, along with the absence of copulation in non-productive pairings, suggest that mate compatibility influences reproductive success in this breeding program (Petit et al. 2024). In the Columbia Basin Pygmy Rabbit breeding program, incorporating mate choice led to improved productivity through increased conception rates and litter sizes (Martin and Shepherdson 2012). Incorporating a similar approach may reduce the occurrence of unproductive pairings in the NEC breeding program.

An important consideration in effectively implementing mate choice is an understanding of the mating system and reproductive cycles of a species, yet little is known about NEC reproductive biology. Like other leporids, NECs have short gestation (31 to 32 d; Petit et al. 2024) and rapid neonatal growth, allowing for multiple litters per season. However, leporids vary in the number of mates they have in a season. Some may be monogamous (white-sided jackrabbits, *Lepus callotisi*; Best and Henry 1993), while some, for example, the Plateau Pika (*Ochotona curzoniae*) exhibits monogamous, polygynous, polyandrous, or polygynandrous mating strategies across different individuals (Dobson et al. 2000). Unfortunately, little is known about the mating systems and mating strategies of NECs. Rabbits in the genus *Sylvilagus* are thought to typically engage in a promiscuous mating system because males may be unable to monopolize multiple females due to their range size and dispersion (Cowan and Bell 1986). The closely related Eastern Cottontail (*S. floridanus*) forms male breeding groups that surround several dominant females (Marsden and Holler 1964), suggesting a polygynandrous mating system. Observations are unavailable for wild NECs, in part due to their cryptic nocturnal behavior in dense early successional habitats. Female NECs at RWPZ mate with different males throughout the breeding season and have multiple litters (Brown and Puccia 2022) and are thought to exhibit post-partum estrous and induced ovulation like eastern cottontails (Casteel 1967). While multiple paternity for a single litter is common among leporids, for example, Snowshoe Hare (*Lepus americanusi*; Burton 2002) and Columbia Basin Pygmy Rabbit (Falcón et al. 2011), this clear indicator of a promiscuous mating system has not yet been observed in NECs. In the conservation breeding program at RWPZ, females paired with 2 males consecutively did not mate with the second male if they had already copulated (Petit et al. 2024). The limited available evidence of the importance of mate compatibility suggests that female mate choice could be an important reproductive strategy in NECs.

The goal of this study is to use an experimental approach to investigate the impact of mate choice on the reproductive success of NECs in the conservation breeding program at RWPZ, and to validate behavioral proxies of preference that can be used to inform future reproductive management of the program. Based on existing information on NEC behavior, females are expected to perform varying frequencies of 1 or more behavioral proxies of preference toward different potential mates in a preference test trial. This variation is expected to then predict reproductive success in subsequent pairings, indicating that mate choice is an important reproductive strategy.

## Methods

### Study site and animals

Wild-caught NECs (*n *= 13; 8 females and 5 males) housed at RWPZ for the ongoing breeding program were used in this study. These rabbits originated in Connecticut (*n *= 6), Massachusetts (*n *= 5), and Maine (*n *= 2). Rabbits were identified using ear tags (left ear for female, right ear for male) that were visible during night and day observations due to the reflective nature of the metal. Rabbit pairs were housed in 4 covered outdoor pens (2.13 m by 2.74 m) enclosed with a wood wall surrounded by fencing. Two infrared cameras were installed on opposite walls in all 4 pens to provide continuous 360° footage and visibility of all interaction areas. Each rabbit had access to hutches and water dispensers. Rabbits were given an ad libitum diet of commercial rabbit feed, greens, and vegetation for browsing. Pens were serviced daily by zoo personnel to remove feces, refresh food and water, and perform any other husbandry tasks. When not paired in the outdoor pens, where all observations for this study occurred, rabbits were housed in indoor laboratory cages. American Society of Mammalogists guidelines were followed (Sikes et al. 2016), and University of Rhode Island Institutional Animal Care and Use Committee approval was obtained (Protocol #AN2122-014).

### Preference test

**Fig. 1:**
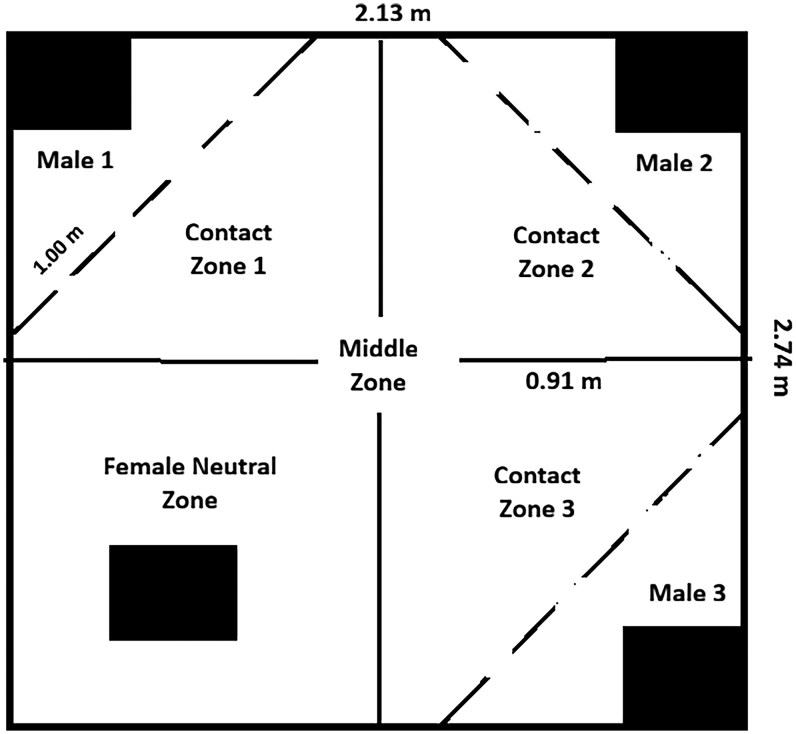
Representation of the mate choice pen setup for New England Cottontail (*Sylvilagus transitionalis*) at Roger Williams Park Zoo in 2022. Black squares represent where hiding structures were located. Dotted black lines represent the mesh barriers, and solid black lines represent solid wooden visual barriers.

Between 30 March 2022 and 11 April 2022, each female spent 24 h with 3 males simultaneously in a specially designed pen (Fig. 1). Males were randomly selected from the 5 available; each male was used in the preference test between 3 and 7 times. Three males each occupied 1 corner of a rectangular pen, with the fourth corner acting as a “neutral zone” for the female. Male areas were separated from the female areas using a 1.5 m × 1.0 m mesh barrier. This barrier allowed female rabbits to visually and olfactorily interact with each male separately. Males were unable to see one another due to horizontally placed wooden barriers. Male vision was obscured as rivalry may influence their behavior, and choice may be hard to distinguish from the resulting competition (Dougherty 2020). Only 1 female was present at any given time during the approximately 24 h preference assessment test, during which she had unlimited access to the neutral zone and the 3 “contact zones” containing the male enclosures, which extend 0.91 m outward from the mesh barrier separating the male and female. Between female introductions, males were rotated to different contact zone enclosures to control for location preference by the females, unrelated to the identity of the male present.

Female preference was assessed using several proxy indicators: visit frequency, time spent in each contact zone, urination frequency within zones, and proximity to or investigation of the mesh barrier. Using BORIS event-logging software (Friard and Gamba 2016), 2 observers analyzed 192 h of video with continuous sampling to quantify the number of visits and time spent in each of the 3 contact zones and the neutral zone (Supplementary Data SD1). A female was considered in a contact zone once she entered the area defined by the dividers (Fig. 1). Additionally, instantaneous sampling at 5 min intervals—conducted by 3 observers—was used to record visible urination spots in each zone and whether the female was near or investigating the male barrier (Table 1).

**Table 1:** Ethogram for the preference test trials (P) and breeding pairings (B) comprised of New England cottontails (*Sylvilagus transitionalis*) at Roger Williams Park Zoo.

Behavior	Description
**Barrier investigation (P)**	The female’s snout is pressed against the mesh barrier separating the contact zone and the male’s pen.
**Urination (P)**	Number of urination spots left by female NEC. Each zone was individually totaled. Spots were not counted more than once, or if the spots were located underneath a water dispenser.
**Female barrier proximity (P)**	The female is within one-half of a body length from the mesh barrier separating the contact zone from the male’s pen.
**Attempted mount (B)**	Male climbs onto the female while stationary or moving and holds the female’s sides with forelimbs, and pelvic thrusting is absent.
**Mount (presumed copulation) (B)**	Male climbs onto the back of the female while stationary while moving and holds the female’s sides with forelimbs, and pelvic thrusting occurs. Ejaculatory falloff may occur.
**Contact (B)**	Any part of the rabbit’s body physically touches any part of the other rabbit’s body.

### Breeding pairings

Breeding took place from 13 April 2022 to 25 July 2022. Females were randomly paired with 1 male from the mate choice test before preferences were known. Each pair was housed for ∼48 h in 1 of 4 outdoor breeding structures. Afterward, females were moved indoors and monitored for offspring. If no kits were born within 45 d, pregnancy was considered unsuccessful, and the female was paired with the next male. If kits were produced, females remained indoors for 2 additional weeks until weaning before being returned to the breeding pen. The number of kits was recorded by keepers within 24 h of birth. This process continued until each female had been paired with all 3 males from the mate choice experiment. Pairings were monitored via 24 h video surveillance, yielding 1,232 h of footage. To ensure all mounting events were captured, the video was analyzed in 2 stages. In Stage 1, 3 observers recorded all instances of physical contact between rabbits. Mounting was considered unlikely in hiding structures due to spatial constraints limiting proper positioning. In Stage 2, 2 observers reviewed each contact event to identify the sex of the initiator and categorize the interaction based on the ethogram (Table 1).

### Interobserver agreement

To assess interobserver agreement for the location of the female during the preference test trial, 4 h of video were coded by both observers, and a pairwise kappa statistic was calculated for each of the 4 zones using HP as the reference observer. The occurrence in each of the 4 zones had excellent agreement (κ > 0.90; Kaufman and Rosenthal 2009). To assess interobserver agreement for urination, female proximity to the barrier, and barrier investigations, all 3 observers coded the same 4 h of video, and an effective percent agreement was calculated. Percent agreement for all behaviors was ≥89% for both observers relative to the reference observer (HP; Supplementary Data SD2).

Interobserver agreement for the observation of contacts during the breeding trials was determined using 2 h of video containing 170 contacts. Once again, HP was utilized as the reference observer, and the individual percent agreement scores were calculated (Supplementary Data SD3). The average interobserver percent agreement was 77.22% for all pairwise comparisons, including 100% of the occurrences of mounting. The second stage observer had 99.07% agreement on sex identification with the reference observer. For attempted mount and mounting, the second observer had a perfect agreement with the reference observer (κ = 1; Kaufman and Rosenthal 2009).

### Data analysis

During the preference test trials, the total time each female spent in the different areas of the mate choice pen was totaled and then divided by total observation time to give a proportion of time spent in each area of the mate choice enclosure. In addition to the proportion of time, the frequency of visits per hour to each of the male contact zones was calculated by totaling the number of visits and dividing by the total hours observed. Urination, barrier investigation, and female proximity proportions were calculated for each of the 3 males the female was exposed to during the mate choice experiment. The proportion of urine spots left in proximity to a male was calculated by dividing the sum of urination spots in each individual male contact zone by the total urination spots in each of the zones of the pen. Barrier investigation and proximity proportions were calculated by totaling the number of barrier investigations and proximity in 1 contact zone and dividing by the total number of scan intervals.

To assess whether urination correlated with time spent in each male contact zone, urination proportions were plotted against the percentage of time females spent in those zones. Linear regression was used to test if the slope differed significantly from 1, with urination proportion as the outcome and time proportion as the predictor. If the confidence interval (CI) of the model includes 1, the slope is not significantly different, suggesting that urine spots were deposited randomly based on the time the female spent in each zone.

Hourly frequencies of behavior observed during the breeding trial (attempted mounts, mounts, and other contacts) were calculated. Contact behaviors were further analyzed by calculating the proportion initiated by males and females within each pair.

### Statistical modeling and linking preference with reproductive success

To test if male identity was associated with female choice, a generalized linear mixed-effect model (GLMM; Bates et al. 2015) was created with the “lme4” package in R (R Core Team 2021). GLMM was selected for its ability to handle repeated measures and account for random effects. This model described any variation in female preference by assessing whether identification of the male (a fixed effect) predicted the amount of time females spent in the contact zone associated with that male. Contact zone time was used as the sole proxy to focus on whether male ID, rather than zone location, influenced female behavior. Female ID and contact zone were included as random effects to account for repeated observations and individual variation. Covariate significance was evaluated using ANOVA to compare models with and without the random effects. Profile likelihood CIs were calculated for the standard deviation of the random intercept (theta), which indicates if individual female ID significantly impacts time spent in contact zones.

To test the relationship between measures of reproductive success and proxy preference indicators, the data were separated into 4 categories: individuals who did and did not produce kits (breeding and non-breeding) and individuals who did and did not mount (mounting and non-mounting). Models generated using generalized estimating equation (GEE) models, an extension of GLM (Højsgaard et al. 2006), tested significant differences between the averages of these groupings. Generalized estimating equations are useful when dealing with small sample sizes and dependent clustered data. GEEs avoid violating the independence assumption of GLM by using a working correlation matrix and cluster-robust standard errors (Huang 2022). GEE does not assume a particular distribution of the data, and its simplistic nature avoids overfitting the model (Huang 2022). The means and SDs of the preference proxies were calculated for each group. Dixon’s tests were used to identify potential outliers in attempted mount and mounting frequencies. This test detects values that are statistically distant from the rest of the data, based on the relative gap between the suspected outlier and its nearest data point compared to the full data range (Efstathiou 2006).

Finally, to determine which preference indicators best predicted reproductive outcome, the correlation between potential preference indicators and reproductive measures of success was modeled using GEE. The goal was to assess the correlation between the fixed effects (preference proxies) and reproductive measures of success. To assess potential multicollinearity among predictor variables, a correlation matrix was generated for all predictor variables. If correlation coefficients exceed 0.90, then their effects would be too collinear to include in the models (Nisbet et al. 2018). To minimize potential collinearity arising from the fact that females had to be near the barrier to investigate it, an interaction term between proximity to the mesh and barrier investigation was included in the model. Success measures were dummy coded for each model as 1 (breeding, mounting pairs) and 0 (non-breeding, non-mounting pairs) and clustered by female ID. Whether or not the pairing consisted of a male and female collected from the same state was also dummy-coded and included as a predictor in the models. Pair order was included as a predictor in the model to test if breeding was more or less likely as the season progressed or as the female was paired with more males. Models with and without pair order and state were created to test if these predictors had coefficients significantly different from 0 using ANOVA. Due to the dichotomous nature of the response variable, the family was specified as binomial. Models were then run with either breeding or mounting observations as the response variable and all preference proxies as predictors. The alpha level for statistical significance was set to α < 0.05.

## Results

### Breeding outcomes and summary statistics

In total, 11 out of 24 pairings produced offspring, with a sum production of 48 kits (Supplementary Data SD4). Litter sizes ranged from 1 to 7 kits (*x̄* = 4.75, SD = 2.01). Eleven pairings were comprised of individuals from different states, of which 6 reproduced (54.55%), and 13 pairings contained individuals from the same state, of which 5 reproduced (38.46%).

### Male ID associated with female behavior during the preference test

In general, females showed behavioral variation in the preference pen (Table 2). Time spent in the contact zones varied significantly by male ID (Table 3) but not contact zone number (χ^2^_(1)_ = 0.058; *P *= 0.81). This pattern indicated that the identity of the male in the contact zone, not the location of the zone, influenced female visitation times. Some males had stronger effects on time spent in the contact zone than others (Table 3), and the random intercept suggests that the specific female in the pen significantly influenced the results (95% CI = 0.26 to 1.23). Urination location was random (CI = –0.49 to 1.64, *P *= 0.28) and was instead negatively related to the amount of time spent in the zone (slope = –0.57).

**Table 2:** The average frequency (visits per hour) and proportion of preference proxies (occurrences out of total number of scans) and 1 SD for the most and least visited male New England cottontails (*Sylvilagus transitionalis*) in the preference test trials conducted at Roger Williams Park Zoo (*n *= 8 female NECs).

	Most visited male		Least visited male	
Potential preference proxies	Mean	SD	Mean	SD
**Proportion of time in contact zone**	0.175	0.127	0.055	0.052
**Proximity to barrier proportion**	0.167	0.141	0.041	0.026
**Barrier investigation proportion**	0.104	0.105	0.022	0.014
**Urination spots proportion**	0.5432	0.176	0.140	0.077
**Visit frequency**	9.364	3.663	4.938	2.903

**Table 3:** Summary of GLMM model of male ID and the proportion of time female New England cottontails (*Sylvilagus transitionalis*; *n *= 8) spent in the contact zones in the preference test trials conducted at Roger Williams Park Zoo.

Predictors	Odds ratios	Confidence interval	*P*
**(Intercept)**	0.18	0.10 to 0.33	**<0.001**
**Male [400744]**	0.43	0.20 to 0.94	**0.034**
**Male [400745]**	0.20	0.07 to 0.63	**0.005**
**Male [400746]**	0.37	0.13 to 1.03	0.058
**Male [400749]**	0.52	0.22 to 1.21	0.127
**Random effects**			
**σ^2^**	3.29		
**τ_00 female_**	0.35		
**ICC**	0.10		
***N*_Female_**	8		
**Observations**	24		
**Marginal *R* ^2^/conditional *R* ^2^**	0.077/0.167		

Bolded values indicated significant difference (*P *≤ 0.05).

### Behavioral differences in breeding behavior between breeding and non-breeding pairs

The mean contact rate was significantly higher in pairings that mounted than in pairings that did not (SE = 1.981; χ^2^ = 5.56; *P *= 0.018; Supplementary Data SD5). There was also a tendency for females to initiate contact more often (62.60% ± 19.63% of contacts) than males. Attempted mounts occurred in 17 (71%) of the pairings, but mounting occurred in only 13 (54%) of all pairings. Of these 13 pairings, 2 did not result in kit production.

### Preference indicators and predicting reproductive outcomes

**Fig. 2:**
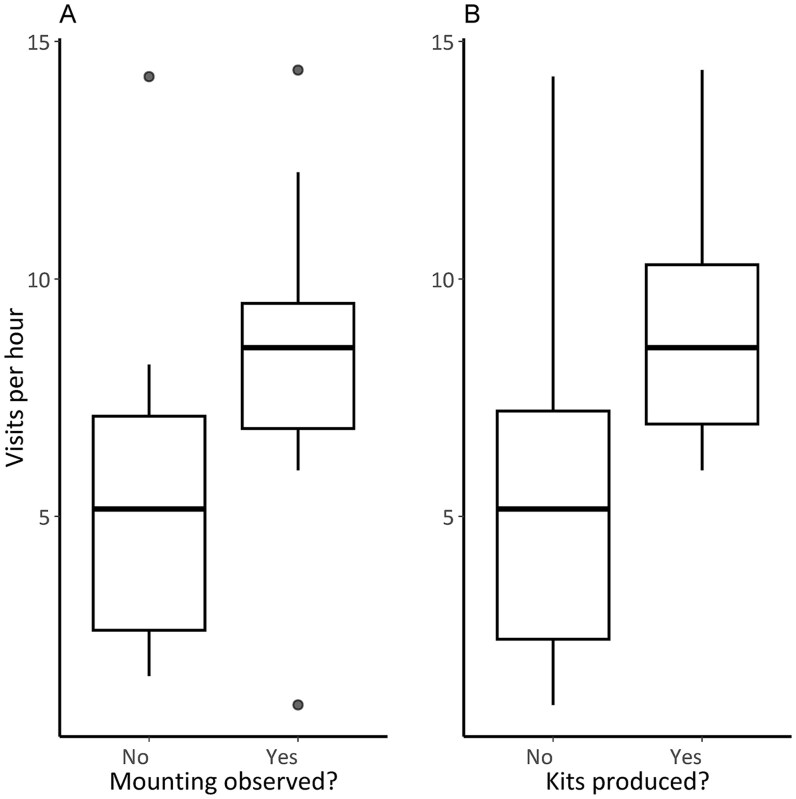
The median and interquartile range of the frequency in which female New England cottontails (*Sylvilagus transitionalis*) at Roger Williams Park Zoo visited males during the preference test trials between mounting (*n *= 13) and non-mounting (*n *= 11) pairs (A) and breeding (kits produced; *n *= 11) and non-breeding (*n *= 13) pairs (B).

Potential preference indicators were not correlated and were thus all included in statistical models (all correlation coefficients < 0.6). The state of origin (χ^2^ = 0.228; *P *= 0.63) and pair order (χ^2^ = 1.48; *P *= 0.65) were not significant predictors of reproductive outcomes and thus were removed to improve the fit of the GEE models. The pairing of male 745 and female 741 was identified as a significant outlier for mounting (*Q *= 0.9; *P *< 0.2 × 10^−15^) and attempted mounts (*Q *= 0.9; *P *< 0.2 × 10^−15^) and was thus removed from the model. No other pairing mounted more than 16 times, but pair 745 to 741 mounted 131 times. While no preference indicators significantly predicted the production of kits, visit frequency significantly predicted the occurrence of mounting (Table 4; Fig. 2; Supplementary Data SD5).

**Table 4:** Summary of GEE models without “state” and “pair order” predictors to find the correlation between proxies for preference and reproductive success measures (if pairings mounted or produced offspring) for New England cottontails (*Sylvilagus transitionalis*) at Roger Williams Park Zoo (*n *= 8, 23 observations).

	Mounting			Kit production		
Predictors	Odds ratios	Confidence interval	*P*	Odds ratios	Confidence interval	*P*
**Intercept**	0.01	0.00 to 2.13	0.088	0.10	0.01 to 1.79	0.118
**Visit frequency**	5.17	1.01 to 26.59	**0.049**	1.64	0.74 to 3.65	0.224
**Urination**	0.95	0.87 to 1.04	0.280	1.00	0.95 to 1.06	0.896
**Proportion of time in contact zone**	1.06	0.80 to 1.41	0.669	0.83	0.61 to 1.13	0.233
**Barrier investigation**	0.23	0.05 to 1.11	0.067	0.72	0.27 to 1.94	0.519
**Barrier proximity**	0.94	0.48 to 1.86	0.869	1.18	0.64 to 2.17	0.602
**Barrier investigation × barrier proximity**	1.01	0.96 to 1.07	0.607	1.00	0.97 to 1.03	0.798

Bolded numbers indicate a significant correlation (*P *≤ 0.05).

**Fig. 3:**
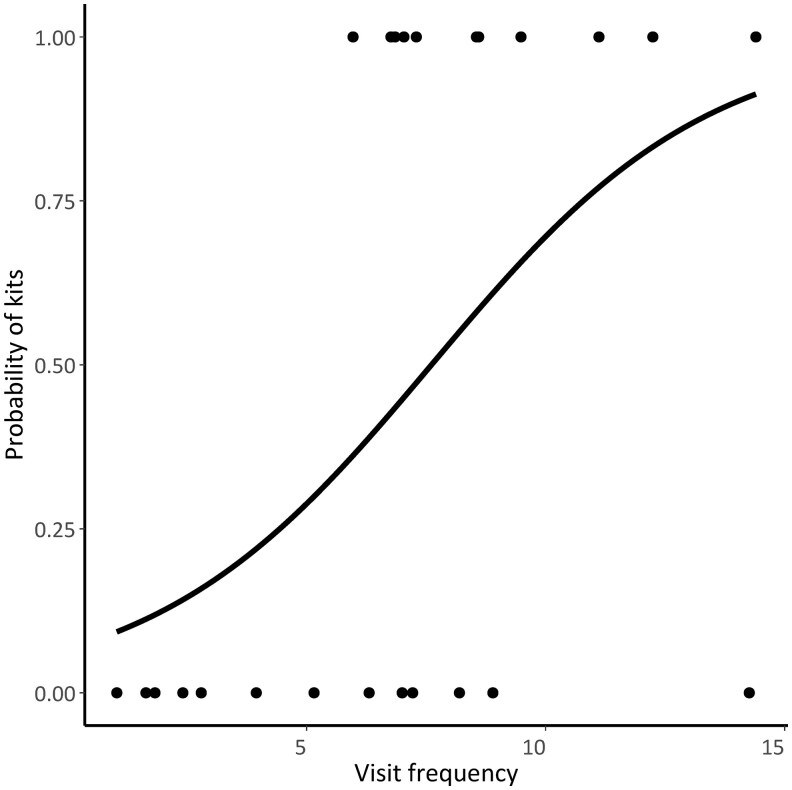
Relationship between visit frequency of female New England cottontails (*Sylvilagus transitionalis*; *n *= 8) to males (*n *= 5) during the preference test trial at Roger Williams Park Zoo and offspring production (kits produced = 1, no kits produced = 0).

Pairings consisting of the male that was visited the most during the preference test were 2.5 times more likely to produce offspring than those containing the least visited males. While all males sired at least 2 litters, only those males that were visited 6 or more times per hour during the preference test later bred with that female. Males visited at least 6 times per hour mounted that female in 68.8% of pairings and sired offspring with that female in 62.5% of pairings (Fig. 3). Female preference did not appear to be universal; all males that were visited 6 or more times per hour by 1 female were also visited less than 6 times per hour by at least 1 other female.

## Discussion

Female NECs in this study demonstrated mate preferences and these preferences influenced reproductive outcomes. Females did not react to all males during the preference test equally, and the number of visits was significantly correlated with mounting when later paired with that male. Visit frequency during a preference test trial can thus be used to identify pairings most likely to be productive. Although the mechanism driving these preferences is unknown, the observation that preference indicated during the trial significantly predicts breeding success months later suggests that females are consistent in preferences and behave accordingly. By increasing the likelihood that each pairing will produce kits, incorporating mate choice opportunities via the preference trial designed for this study will greatly increase the conservation impact of the breeding program.

### Preference proxies

Despite measuring 5 different potential proxy behaviors that have been useful in studies of mate choice in other species, including leporids, only visit frequency was positively correlated with a measure of reproductive success. Interestingly, the amount of time females spent with each male and the proportion of scans proximal to the barrier separating them had low predictive power on the reproductive outcomes. This observation of short, frequent interactions is consistent with field mate choice studies on the American Pika (*Ochotona princeps*), in which the number of encounters between males and females during the breeding season was higher than expected, but the duration of these encounters only accounted for 2.6% of the total observation time (Brandt 1989). In the closely related Eastern Cottontail, both males and females expand their home range during the breeding season, suggesting active searching for mates (Haugen 1942); increased visit frequency in zoo NECs may similarly reflect the active search for males. If wild NECs also have mate preferences, then more active females that encounter a larger variety of males may have better options for mate selection. While visit frequency predicts the occurrence of copulation, the lack of a significant relationship between this proxy for preference and kit production may be attributed to the confounding potential for non-fertile mating or pregnancy loss. The reason that 2 pairings copulated but did not produce kits could not be determined in this study; developing minimally invasive means for detecting pregnancy could be helpful for program management. Continued observations may reveal alternative potential proxies that more accurately predict kit production.

Female preference is dependent on their assessment of male characteristics. The male traits that are evaluated during these visits are unknown but could include morphological, behavioral, or physiological characteristics. Each male was preferred by at least 1 female, suggesting that universal female preference for a morphological trait such as body size is unlikely. Female NECs may assess male behavior (Spritzer et al. 2005), but a redesign of the pen would be required to measure male behavior as they were obscured from the view of the cameras in this study. Olfactory cues can be another important mechanism of mate choice (Parrott et al. 2019). Lagomorphs would be predicted to use these cues; they rely heavily on scent marking to define territories, and urine contains olfactory information about the age, sex, and social rank of an animal (Bell 1980). However, barrier investigation behavior, when olfactory investigation would conceivably occur, was negatively correlated with the observation of mounting. Due to well-developed olfactory senses in lagomorphs, females may have been able to smell each of the males without making direct contact with the mesh. Similarly, the proportion of urination spots left by the female in the contact zones was not correlated with reproductive success of a pair. Female investigation of male scent may be a better predictor of preference than female urination near males, but male urination was unable to be observed in this study. To further test the possibility of olfactory signaling in NECs, preference trials could be conducted with just male odor cues.

### Breeding behavior and reproductive success

Considerable behavioral differences were observed between pairings with different reproductive outcomes. Pairings that copulated made physical contact with each other more frequently than those that did not copulate, indicating that these pairings were able to remain within proximity more often. Similar patterns of females remaining in close proximity with preferred mates have been observed in savanna baboons (*Papio cynocephalus*; Bercovitch 1995) and rhesus monkeys (*Macaca mulatta*; Cochran 1979). Female NECs in our study initiated more contacts, in contrast to the breeding behavior of eastern cottontails, in which male breeding groups perform most contacts (Marsden and Holler 1964). Female-initiated contacts may function to displace males or engage with preferred males. In a previous study, female NECs initiated more chases and charges that resulted in decreased proximity and thus fewer interactions, regardless of whether that pairing was successful or not (Petit et al. 2024). Males that are better able to remain in contact range would therefore elicit more female-initiated contacts. The observation that a female initiated significantly more contact with males who sired her offspring in this study suggests that males who maintain proximity may be preferred by the female. The ability of males to consistently remain in the contact zone could be a means for females to select for more agile males (Orbach et al. 2020), a potentially important trait for this cryptic prey species.

Higher female engagement with preferred males and the rarity of mounting behavior in non-breeding pairs suggest that pre-copulatory sexual selection is an important reproductive strategy in this species. However, the necessarily small sample size, lack of data on age and breeding experience from individuals before entering the breeding program, and the potential for outlying variation in behavior precludes broader conclusions. For example, female 741 may not have been in breeding condition when paired with male 745. The female rarely attempted to flee from this persistent male, resulting in extremely high frequencies of mounting behavior that necessitated the removal of this pair from statistical modeling. The small sample size also inhibited the ability to present females with a known range of male phenotypes for the preference test. While the NECs in this study originated from different states across New England—presumably increasing genetic diversity—it is possible that the phenotypes of the available males did not vary significantly, or that females in this study were all similar or dissimilar to the population mean of preferences by chance. While increasing the sample size would lead to stronger conclusions, the availability of animals for study is restricted by the declining populations of NECs in the wild.

### Applications to the management of the breeding program and wild populations

This study has expanded the current knowledge of NEC reproductive strategies, enabling breeding program management that could maximize offspring production. Extrapolating from the findings of this study, if females are only paired with males that visited 6 or more times per hour during the preference test trial, overall breeding success would double when compared to random pairings. This increased offspring production further justifies the future removal of individuals from the wild to support the program, which has provided a source of NECs to establish self-sustaining populations in the wild (Brown and Puccia 2022). Additionally, wild populations are likely experiencing decreases in genetic diversity due to fragmentation (Fenderson et al. 2011). As individual NECs in the breeding program originate from habitat patches throughout New England, the reintroduction of captive-bred offspring may be one of the few viable options to increase genetic diversity in those populations. While incorporating mate choice improved kit production, kit survivability remained low (Supplementary Data SD4). If this factor could be addressed, the increased kit production would ensure that the breeding program would create a substantial impact on NEC populations.

Understanding the role of mate choice in NEC reproduction is also critical for developing conservation strategies for wild populations. Females in this study did not mate with non-preferred males despite only 1 male being present inside each breeding pen at a time. Females in wild populations may similarly delay copulating with non-preferred mates. Similar results were observed in field studies on pikas, where it was found that despite interacting with multiple males, females only copulated with 1 male each (Brandt 1989). For females to exercise mate choice, individuals must have the ability to make reproductive decisions (Candolin and Wong 2019). However, with declining populations and fragmentation across inhospitable landscapes, females may not encounter a preferred male and forego breeding that season. Additionally, male breeding strategy, driven by female choosiness, may increase mortality. Male cottontails tend to have a greater range during the breeding seasons but have limited dispersal abilities; discontinuous habitats could thus cause an increase in dispersal-related mortality for individuals attempting to interact with more potential mates (Giuntini and Pedruzzi 2023). These factors would indicate that population sex ratios may be an important factor when deciding where to release captive-bred individuals to ensure that female mate choice can occur within that habitat patch. Whether females refrain from mating or selection pressures change so that maladaptive pairings occur more frequently, limitations to mate choice can decrease population viability. Therefore, this behavior should be considered in the overall management of wild NEC recovery efforts.

## Supplementary Material

gyag023_Supplementary_Data

## Supplementary data

**Supplementary Data SD1.** List of females and the males they were exposed to during the preference test. The start date refers to the day the female was placed in the pen. The end date refers to the day the female was removed from the pen.

**Supplementary Data SD2.** Instantaneous sample observer percentage agreement between HP and instantaneous observers.

**Supplementary Data SD3.** Contact observer percentage agreement between HP and contact observers.

**Supplementary Data SD4.** Data for preference proxies and reproductive measures of success listed by pairing (*n* = 24).

**Supplementary Data SD5.** Median behavioral frequencies across all types of New England Cottontail (*Sylvilagus transitionalis*) pairings at Roger Williams Park Zoo.
