# Supplementary material for: Determining behavioral proxies of preference: mate choice and the New England Cottontail (Sylvilagus transitionalis)
Source: J Mammal. 2026 Jul 9;107(4):785–93. doi: 10.1093/jmammal/gyag023 (PMC13416184; doi:10.1093/jmammal/gyag023)
Supplement: gyag023_Supplementary_Data [file gyag023_supplementary_data.zip › Supplementary Data SD1.pdf]

**Supplementary Data S1.** List of females and the males they were exposed to during the preference test. The start date refers to the day the female was placed in the pen. The end date refers to the day the female was removed from the pen.

| Female | Male 1 | Male 2 | Male 3 | Start Date | End Date |
|---|---|---|---|---|---|
| 741 | 400740 | 400744 | 400745 | 4/1/2022 | 4/2/2022 |
| 742 | 400740 | 400744 | 400745 | 3/30/2022 | 3/31/2022 |
| 743 | 400740 | 400744 | 400745 | 3/31/2022 | 4/1/2022 |
| 747 | 400746 | 400749 | 400745 | 4/2/2022 | 4/3/2022 |
| 750 | 400746 | 400749 | 400740 | 4/4/2022 | 4/5/2022 |
| 753 | 400744 | 400740 | 400746 | 4/8/2022 | 4/9/2022 |
| 754 | 400749 | 400740 | 400744 | 4/9/2022 | 4/10/2022 |
| 755 | 400749 | 400740 | 400744 | 4/10/2022 | 4/11/2022 |
