# Supplementary material for: Determining behavioral proxies of preference: mate choice and the New England Cottontail (Sylvilagus transitionalis)
Source: J Mammal. 2026 Jul 9;107(4):785–93. doi: 10.1093/jmammal/gyag023 (PMC13416184; doi:10.1093/jmammal/gyag023)
Supplement: gyag023_Supplementary_Data [file gyag023_supplementary_data.zip › Supplementary Data SD2.pdf]

Supplementary Data S2. Instantaneous sample observer percent agreement between HP and instantaneous observers.

Observer	Urination spots	Barrier investigation	Mesh proximity
Obs 1.	100%	100%	100%
Obs 2.	96%	100%	89%
