# Supplementary material for: Determining behavioral proxies of preference: mate choice and the New England Cottontail (Sylvilagus transitionalis)
Source: J Mammal. 2026 Jul 9;107(4):785–93. doi: 10.1093/jmammal/gyag023 (PMC13416184; doi:10.1093/jmammal/gyag023)
Supplement: gyag023_Supplementary_Data [file gyag023_supplementary_data.zip › Supplementary Data SD3.pdf]

Supplementary Data S3. Contact observer percent agreement between HP and contact observers.

Observer	Percent agreement
Obs 2.	83.12%
Obs 3.	75.32%
Obs 4.	75.16%
Obs 5.	75.29%
