# Supplementary material for: Determining behavioral proxies of preference: mate choice and the New England Cottontail (Sylvilagus transitionalis)
Source: J Mammal. 2026 Jul 9;107(4):785–93. doi: 10.1093/jmammal/gyag023 (PMC13416184; doi:10.1093/jmammal/gyag023)
Supplement: gyag023_Supplementary_Data [file gyag023_supplementary_data.zip › Supplementary Data SD5.pdf]

Supplementary Data S5. --- Median behavioral frequencies across all types of New England cottontails (*Sylvilagus transitionalis*) pairings at Roger Williams Park Zoo.

	Breeding		Non-Breeding		Mounting		Non-Mounting	
Behaviors	*Median*	*IQR*	*Median*	*IQR*	*Median*	*IQR*	*Median*	*IQR*
Physical contact	1.3	0.19-4.29	0.09	0.05 – 0.46	1.52	0.32-6.96	0.09	0.05 – 0.46
Attempted mounts	0.15	0.10-0.43	0.00	0.00-0.115	0.14	0.06-0.31	0.00	0.00-0.02
Mounts	0.13	0.06 - 0.25	0.00	0.00-0.00				
