# Supplementary figures and images for: Determining behavioral proxies of preference: mate choice and the New England Cottontail (Sylvilagus transitionalis)
Source: J Mammal. 2026 Jul 9;107(4):785–93. doi: 10.1093/jmammal/gyag023 (PMC13416184; doi:10.1093/jmammal/gyag023)

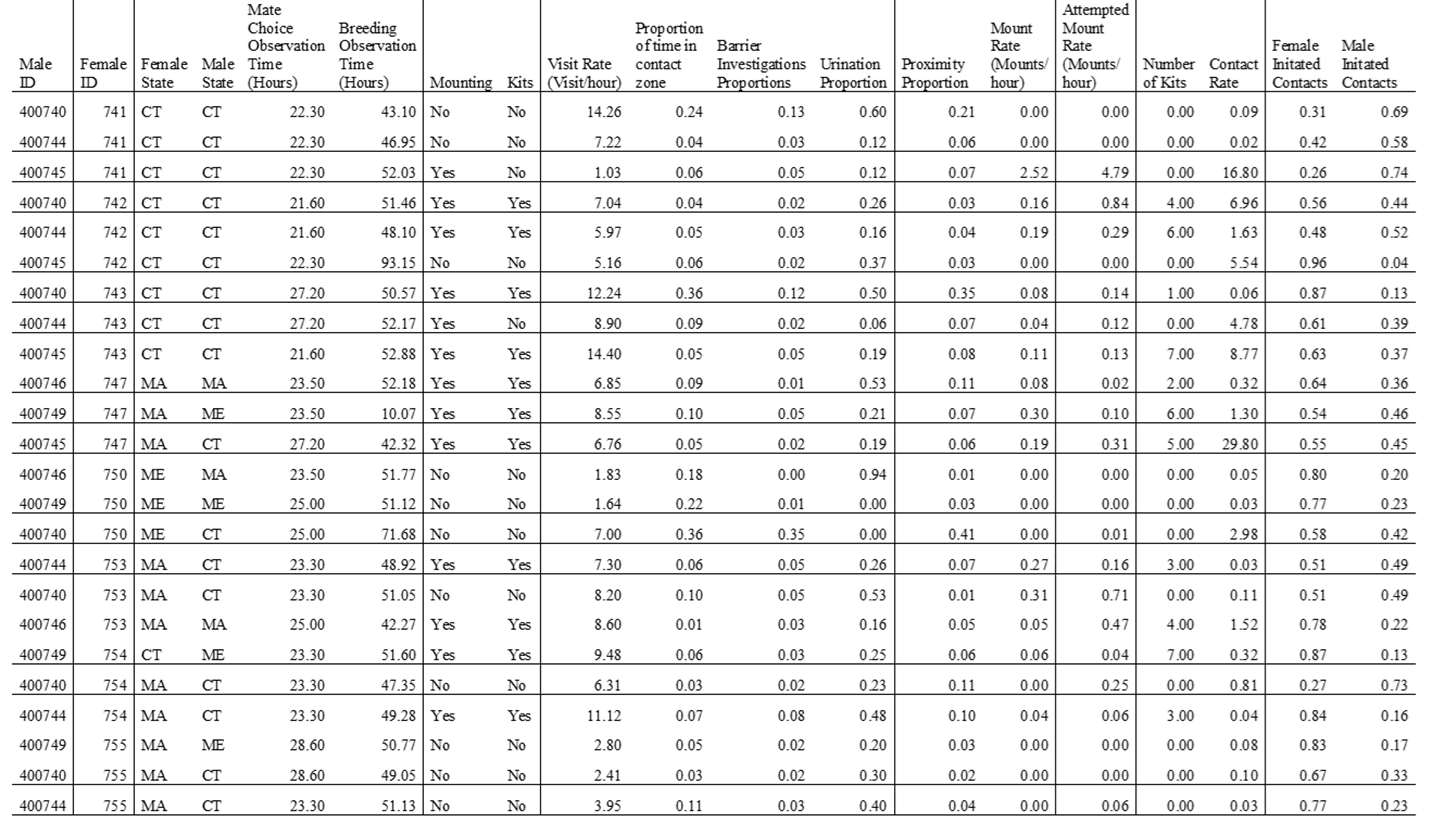

Supplement: gyag023_Supplementary_Data [file gyag023_supplementary_data.zip › Supplementary Data SD4.tif]
